# Narrow-Band Imaging for the Detection of Early Gastric Cancer Among High-Risk Patients: A Systematic Review and Meta-Analysis

**DOI:** 10.3390/medicina61091613

**Published:** 2025-09-06

**Authors:** Magdalini Manti, Paraskevas Gkolfakis, Nikolaos Kamperidis, Alexandros Toskas, Apostolis Papaefthymiou, Georgios Tziatzios, Ravi Misra, Naila Arebi

**Affiliations:** 1 Gastroenterology Unit, St Mark’s Hospital, Acton Ln, London NW10 7NS, UK; manti.magdalini@gmail.com (M.M.); nkamperidis@nhs.net (N.K.); a.toskas@nhs.net (A.T.); rmisra@nhs.net (R.M.); naila.arebi@imperial.ac.uk (N.A.); 2 Hepatogastroenterology Unit, Second Department of Internal Medicine-Propaedeutic, Attikon University Hospital, Rimini 1, Chaidari, 12462 Athens, Greece; 3 Department of Gastroenterology, General University Hospital of Larissa, 41334 Larissa, Greece; appapaef@hotmail.com; 4 Department of Gastroenterology, “Konstantopoulio-Patision” General Hospital of Nea Ionia, 14233 Athens, Greece

**Keywords:** gastric cancer, NBI, white-light imaging

## Abstract

*Background and Objectives*: Early gastric cancer (EGC) has an excellent prognosis when detected, yet miss rates during endoscopy remain high. Narrow-band imaging (NBI) enhances mucosal and vascular visualization and is increasingly used, but its benefit over white-light imaging (WLI) in high-risk patients is uncertain. This study aimed to compare NBI with WLI for the detection of gastric neoplasia in patients undergoing gastroscopy. *Materials and Methods*: We conducted a systematic review and meta-analysis of randomized controlled trials (RCTs), registered in PROSPERO (CRD42025649908) and reported according to PRISMA 2020 guidelines. PubMed, Scopus, and CENTRAL were searched up to October 2024. Eligible RCTs randomized adults undergoing gastroscopy for cancer surveillance or red-flag symptoms to NBI or WLI. Data extraction and risk of bias assessment were performed independently by two reviewers. Pooled relative risks (RRs) with 95% confidence intervals (CIs) were calculated using a random-effects model, and certainty of evidence was graded with GRADE. *Results*: From 21 records, 3 RCTs comprising 6003 patients were included. NBI did not significantly increase gastric neoplasm detection compared with WLI (2.79% vs. 2.74%; RR = 0.98; 95% CI: 0.66–1.45; I^2^ = 22%). Focal gastric lesion detection rates (14.73% vs. 15.50%; RR = 1.05; 95% CI: 0.72–1.52; I^2^ = 87%) and positive predictive value (29.56% vs. 20.56%; RR = 1.29; 95% CI: 0.84–1.99; I^2^ = 61%) also showed no significant differences. Risk of bias was high for blinding, and overall evidence certainty was low. In practical terms, both NBI and WLI detected gastric cancers at similar rates, indicating that while NBI enhances visualization, it does not increase the likelihood of finding additional cancers in high-risk patients. *Conclusions*: NBI did not significantly improve gastric neoplasm detection compared with WLI in high-risk patients, though it remains valuable for mucosal and vascular assessment. Larger, multicenter RCTs across diverse populations are required to establish its role in surveillance strategies.

## 1. Introduction

In 2020, gastric cancer ranked as the fifth most frequently diagnosed malignancy and the fourth leading cause of cancer-related mortality globally, accounting for 769,000 deaths—twice as frequent in men [1]. Its 5-year survival rate is approximately 20%, with H. pylori as the main risk factor [2]. However, early gastric cancer (EGC) has a survival rate over 95% [3] and can often be treated endoscopically [4]. It is more prevalent in East Asia and Eastern Europe [2], with a recent rise in cases among adults under the age of 50 globally [5].

The development of gastric cancer involves a chain of mucosal progression from normal, to inflamed and then atrophic areas, which could further lead to intestinal metaplasia (IM), dysplasia and finally to gastric adenocarcinoma [4]. EGC is limited to the mucosa or submucosa (T1), with or without lymph node involvement.

In terms of EGC detection, the miss rates remain relatively high (6–10%) [6]. Conventional white-light imaging (WLI) shows variable sensitivity (67.9–94.3%) [7], while high-definition WLI improves detection moderately, with 74.6% sensitivity, 94% specificity, and 88% accuracy [8]. However, via the use of conventional WLI along with magnifying endoscopy (ME) lesions can further distinguish between cancerous and benign [7]. Currently, ME, using lens systems, can enlarge images from 1.5× up to 150× and generate high-resolution visuals with pixel densities reaching 850,000, enabling the detection of structures as small as 10 to 71 μm in diameter [9,10]. Apart from the conventional ME, digital magnification has also been introduced, also called dual or “near focus’’ (NF) which provides up to 45× magnification [11].

Based on the recently updated MAPS III guidelines [4], high-quality endoscopy including virtual chromoendoscopy (VCE) should be performed for diagnosis, screening and staging both in precancerous conditions and in EGC. Narrow-band imaging (NBI) (Olympus Medical Systems, Tokyo, Japan), developed by Sano et al. in 1999 [12], is the most widely used optical VCE technique. It enhances mucosal surface and microvascular contrast by filtering white light into narrow bands [13,14,15,16]. The VS (vessel and surface) classification helps diagnose EGC by assessing lesion borders, vascular patterns, and surface structures [17]. NBI image quality depends on the endoscopy system, with three generations available. The latest, 3G-NBI (third generation) (EVIS X1), uses a five-color LED for improved brightness [13]. Combining NBI with ME enhances lesion detection [18], particularly with the NF-NBI mode [19,20]. Alternative techniques include blue-laser imaging (BLI) (Fujifilm Co., Tokyo, Japan), linked-color imaging (LCI) (Fujifilm Co., Tokyo, Japan), and dye-spray chromoendoscopy [4]. Artificial intelligence (AI)-assisted endoscopy is also an emerging field, with convolutional neural networks trained to identify subtle mucosal changes.

Red-flag symptoms that prompt the referral for a gastroscopy include unexplained weight loss, dyspepsia over the age of 55, dysphagia, hematemesis, anemia [21]. Apart from that, surveillance should be performed for patients with a previous diagnosis of IM, gastric atrophy (GA), low-grade dysplasia and post-endoscopic resection.

The rationale for this review is based on the need to clarify the role of NBI in the detection of early gastric cancer, given existing conflicting evidence.

The primary objective of this study was to was to compare NBI with WLI for the detection of gastric neoplasia in high-risk patients.

## 2. Materials and Methods

This study was conducted in accordance with the PRISMA 2020 (Preferred Reporting Items for Systematic Reviews and Meta-Analyses) guidelines [22] and all authors complied with the protocol (Table A1). Additionally, the protocol was registered with PROSPERO under the identifier CRD42025649908.

### 2.1. Eligibility Criteria

We structured the meta-analysis according to the validated PICO framework [23]. The primary research question focused on comparing NBI with WLI in patients with a history of gastric cancer, candidates for endoscopic surveillance via gastroscopy or red-flag symptoms. Patients with known malignancy were studied to evaluate the presence of synchronous EGC which might have been missed by the initial referring hospitals. The investigation of progression of pre-malignant conditions, such as gastric atrophy and intestinal metaplasia [24], was not included in our study.

This meta-analysis included only randomized controlled trials (RCTs) involving adult participants. Eligible patients had undergone gastroscopy for one of the following indications: post-gastric cancer surveillance, preoperative evaluation, or investigation of red-flag symptoms. During the procedures, participants were assigned to undergo examination either with narrow-band imaging (NBI) or white-light imaging (WLI).

Only RCTs reporting specific outcomes—particularly the gastric neoplasm detection rate (GNDR)—were included in the synthesis. Studies published in languages other than English were excluded. Additional exclusion criteria included inadequate reporting of participant characteristics or conclusions, as well as non-randomized study designs.

### 2.2. Study Endpoints—Definitions

The primary endpoint was the comparative GNDR, calculated by dividing the number of gastroscopies detecting gastric cancer by the total number of procedures and multiplying by 100.

Secondary outcomes were the focal gastric lesion detection rate (FGLDR) -including adenomas, intestinal metaplasia and gastric cancer—which were calculated similarly. Additionally, we studied the positive predictive value (PPV) of the method. The latter refers to the proportion of patients who were correctly diagnosed with gastric cancer among those who had gastric lesions using either NBI or WLI. This was verified by histology post-esophagogastroduodenoscopy (OGD). Comparisons between these technologies for these outcomes were also performed.

### 2.3. Search Methodology

A structured search of the literature was conducted by two independent reviewers (MM and AT) between September and October 2024. Searches were performed across Medline (via PubMed), Scopus, and the Cochrane Central Register of Controlled Trials. The strategy included a combination of Medical Subject Headings (MeSH) and free-text terms: “narrow band imaging” or “NBI,” “white light imaging” or “WLI,” “gastric cancer,” “gastric neoplasia,” “stomach cancer,” “stomach neoplasia,” and “surveillance.” These were paired using the Boolean operator AND with the term “high risk patients,” also searched as both a MeSH and free-text term. After the electronic search, reference lists of the retrieved studies were manually reviewed to find further eligible studies. All retrieved articles were screened for eligibility, first by one reviewer (MM) and afterwards by two independent senior authors (NK and AT), using the predetermined inclusion criteria. Titles and abstracts of all retrieved records were initially screened, after which the full texts of potentially eligible studies were independently reviewed for inclusion. In instances where multiple publications originated from the same study, only the most recent and comprehensive version was considered. Furthermore, if both parallel-group and crossover designs were reported, only data from the parallel-group trials were included in the analysis.

### 2.4. Data Extraction Process

Two investigators (MM and AT) independently reviewed all eligible studies and extracted relevant data—including study characteristics, participant information, and intervention details—using a standardized data extraction form. A third reviewer (NK) cross-checked the extracted datasets for consistency. Any discrepancies were resolved through discussion, and when necessary, with input from a senior investigator (PG or AP) to reach consensus.

### 2.5. Risk of Bias Assessment

The risk of bias in each included study was independently assessed by three reviewers (MM, AT and AP). In cases where discrepancies arose, these were resolved through consultation with a third investigator (NK). The evaluation was performed using the Cochrane Collaboration’s Risk of Bias Tool [25], which systematically examines key methodological domains that may influence study validity. These domains include selection bias, performance bias, detection bias, attrition bias and reporting bias. Each domain was judged as presenting a low, high, or unclear risk of bias according to the reviewers’ appraisal.

### 2.6. Analysis Methods

Data analysis was performed using Review Manager (RevMan) version 5.4.1 (The Nordic Cochrane Centre, The Cochrane Collaboration, Copenhagen, Denmark, 2020). For both the primary and secondary outcomes, relative risks (RRs) with corresponding 95% confidence intervals (CIs) were assessed. Forest plots were generated to visually represent the findings. A random-effects model, based on the DerSimonian and Laird method, was applied to synthesize results regardless of the degree of heterogeneity. Statistical heterogeneity across studies was assessed using the I^2^ statistic, with values below 30% considered indicative of low heterogeneity, and those between 30% and 60% representing moderate heterogeneity. We planned sensitivity analyses by sequentially excluding individual studies from the meta-analysis to assess whether any single trial disproportionately influenced the overall effect estimates. We explored potential sources of heterogeneity by examining differences in imaging modality, comparator definitions and study population characteristics. Planned subgroup analyses were limited by the small number of eligible trials.

### 2.7. Grading the Evidence Strength

The quality of the provided evidence was rated based on the GRADE criteria. Two independent researchers (MM and AT) graded inconsistency, risk of bias, indirectness, imprecision and publication bias. Overall quality was deemed very low, low, moderate, or high, using GRADEpro (GRADE Working Group) [25].

## 3. Results

### 3.1. Overview of Included Studies

**Figure 1:**
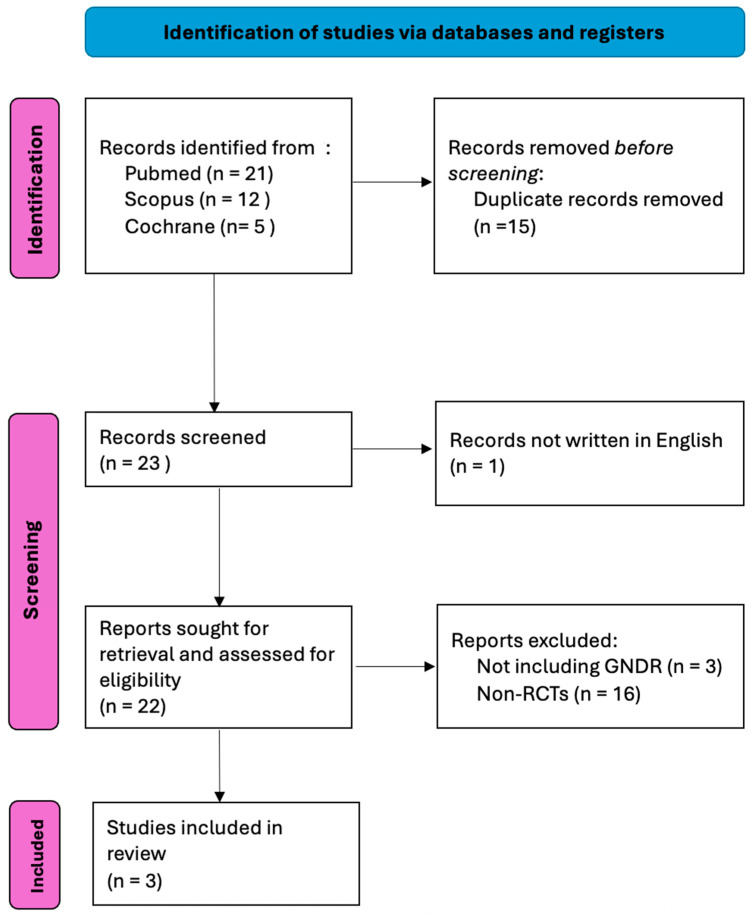
PRISMA flowchart.

A total of 21 unique records were initially identified through the database search. After screening and applying the predefined exclusion criteria, three studies [26,27,28] fulfilled the inclusion criteria and were incorporated into the final meta-analysis. The study selection process is detailed in the PRISMA flow diagram (Figure 1), and the main features of the included studies are summarized in Table 1.

In total, 6003 patients were enrolled, with a balanced female-to-male ratio of 1:1. Patients were over the age of 20 in 2 studies [26,28] and over 50 in 1 study [27]. All the studies were multicenter. Two studies had a single origin (Japan) [26,28], while one study was international including Asia Pacific population from Australia, China, Malaysia, Singapore and Thailand [27]. The included studies recruited patients between 2012 and 2022, with individual study durations ranging from one to three years from initiation to completion.

One study [28] included only patients with a history or active gastric cancer, while 2 studies [26,27] included patients with red-flag symptoms.

Two studies [27,28] utilized 2G-NBI while one study used 3G-NBI [26]. Additionally, all the studies used digital magnification (NF). Among the included studies, two [26,28] utilized high-definition WLI, while one study [27] employed standard-definition WLI.

All procedures were conducted by skilled endoscopists proficient in using NBI. Each gastroscopy began with either WLI or NBI. After completing the examination and documenting findings with the initial modality, endoscopists had the option to further evaluate the stomach using the alternative modality.

All studies enrolled individuals undergoing upper gastrointestinal endoscopy for gastric cancer surveillance and/or screening due to the presence of red-flag symptoms.

### 3.2. Evidence Quality Overview

**Figure 2:**
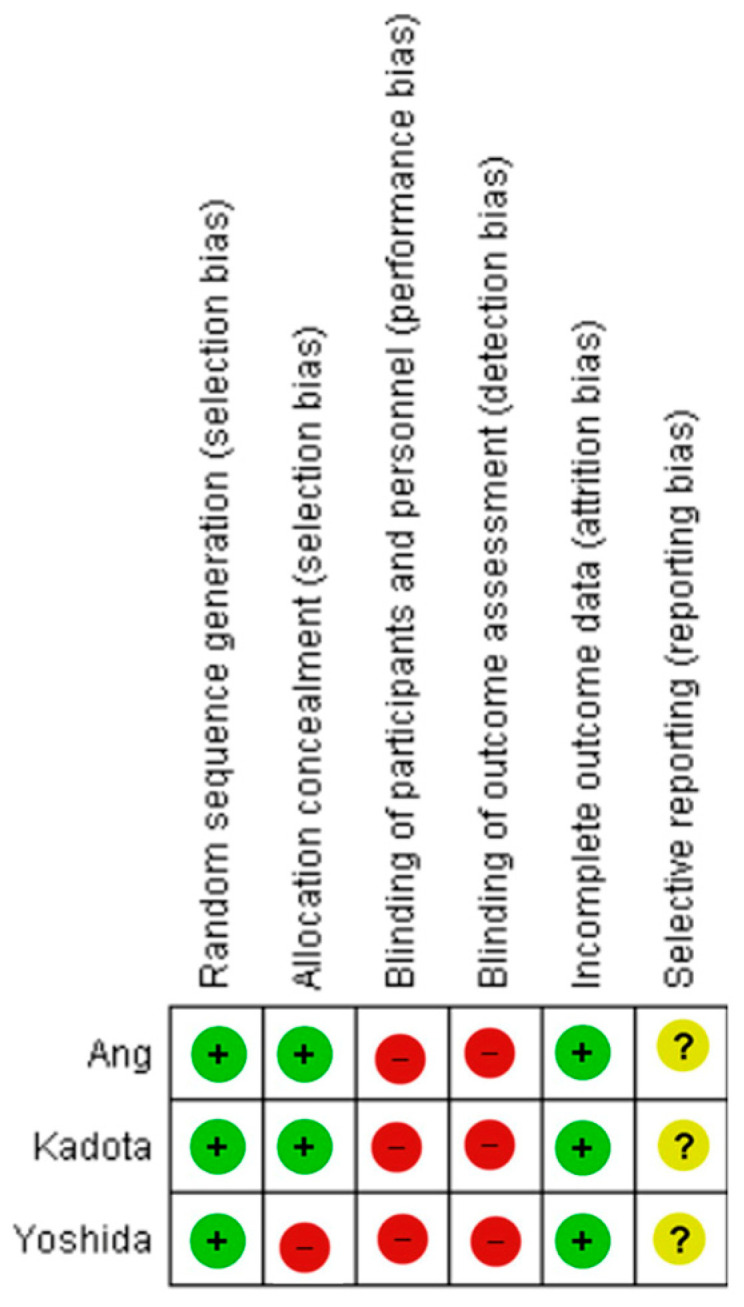
Risk of bias summary of studies [26,27,28].

Figure 2 provides an overview of the risk of bias across the included studies, as assessed using the Cochrane Collaboration’s Risk of Bias Tool. In all cases, physicians were aware of the imaging modality and study outcomes, introducing a potential risk of performance and detection bias. We did not detect evidence of reporting bias across the included syntheses. Still. the small number of included studies precluded formal funnel plot assessment or statistical tests for publication bias. A comprehensive, domain-specific breakdown of the bias assessment is presented in Table A1, Table A2 and Table A3.

### 3.3. Quality of Evidence According to GRADE

The certainty of the evidence supporting the effect estimates was judged to be low overall. Regarding the primary outcome, the evidence was downgraded by two levels: once for risk of bias, due to the inability to blind endoscopists to the intervention, and once for indirectness, as the procedures were conducted by expert practitioners within specialized centers, limiting generalizability. A detailed evaluation of the evidence quality based on the GRADE framework is provided in Table A4.

### 3.4. Primary Endpoint

**Figure 3:**
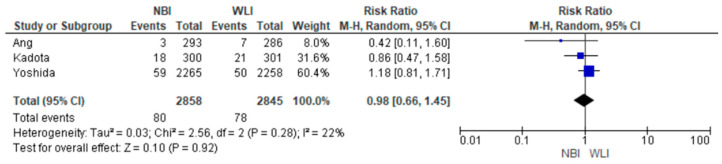
Primary endpoint: GNDR in NBI versus WLI [26,27,28].

**Figure 4:**
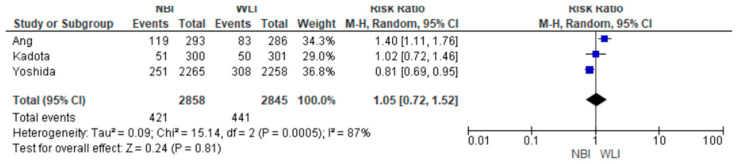
Secondary endpoint: FGLDR in NBI and WLI [26,27,28].

**Figure 5:**
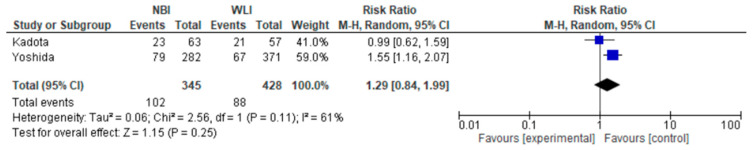
Secondary endpoint: PPV in NBI versus WLI [26,28].

For each included study, we present group-specific summary statistics (numbers of patients with events and denominators) together with study-level effect estimates and their 95% confidence intervals. These data are displayed in the forest plots (Figure 3, Figure 4 and Figure 5), which provide both individual trial results and the pooled synthesis.

Overall, the use of NBI increased GNDR compared to WLI [2.79% vs. 2.74%; RR (95%CI) = 0.98 (0.66–1.45); *p* = 0.92; Ι^2^ = 22%]. However, this change was not statistically significant (Figure 3).

### 3.5. Secondary Endpoints

- Focal gastric lesion detection rates were also not statistically significant between the two groups [14.73% vs. 15.50%; RR (95%CI) = 1.05 (0.72–1.52); *p* = 0.81; Ι^2^ = 87%] (Figure 4).
- Positive predictive value: Data from two studies [26,28] indicated no statistically significant difference in the PPV between NBI and WLI, with reported values of 29.56% vs. 20.56%, respectively (RR = 1.29; 95% CI: 0.84–1.99; *p* = 0.25; I^2^ = 61%) (Figure 5).

**Figure 6:**
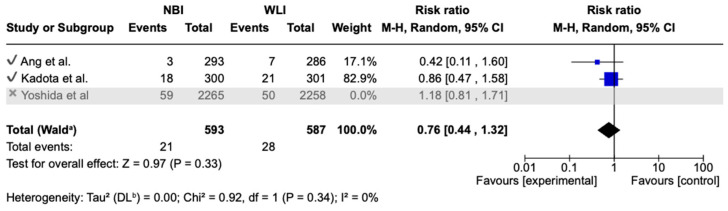
Secondary endpoint: sensitivity analysis in terms of GNDR [26,27,28]. Waldᵃ = pooled total calculated using the Wald test. DLᵇ = DerSimonian–Laird estimator for between-study variance (τ^2^).

Sensitivity analyses were conducted by sequentially excluding each included study to evaluate the robustness of the pooled estimates. Exclusion of the largest trial [28] did not materially alter the effect size for GNDR or secondary outcomes. In detail, in terms of GNDR analysis, the pooled effect shifted slightly in favor of NBI (RR 0.76, 95% CI 0.44–1.32), and statistical heterogeneity was eliminated (I^2^ = 0%). These findings suggest that the conclusions remain robust, although the effect estimate may be influenced by the largest trial (Figure 6).

**Figure 7:**
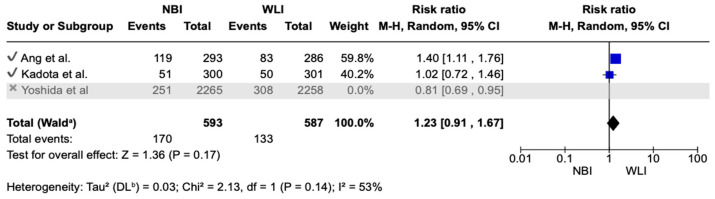
Secondary endpoint: sensitivity analysis in terms of FGLDR [26,27,28]. Waldᵃ = pooled total calculated using the Wald test. DLᵇ = DerSimonian–Laird estimator for between-study variance (τ^2^).

When evaluating FGLDR, the pooled RR shifted to 1.23 (95% CI 0.91–1.67, I^2^ = 53%), with reduced heterogeneity. This suggests that the overall conclusions are not materially altered by the exclusion of the largest study, although the precision of the estimate remains limited due to the small number of included trials (Figure 7). This suggests that the overall conclusions are not materially altered by the exclusion of the largest study, although the precision of the estimate remains limited due to the small number of included trials.

Similarly, omission of either of the smaller studies [26,27] yielded results consistent with the primary analyses, confirming the stability of our findings. Moderate heterogeneity was observed for FGLDR (I^2^ = 87%) and PPV (I^2^ = 61%), while heterogeneity for GNDR was low (I^2^ = 22%). Exploration of potential sources, including endoscope generation and comparator modality, did not explain the observed heterogeneity, and further subgroup analyses were not feasible due to the small number of trials.

## 4. Discussion

To the best of our knowledge, this is the first meta-analysis to directly compare NBI and WLI in patients at high risk for gastric cancer. Although NBI increased GNDR compared to WLI, there was no statistical significance in our study.

From a clinical perspective, these findings suggest that while NBI may improve mucosal and vascular visualization, its routine use does not currently provide a clear advantage in increasing gastric cancer detection rates. For endoscopists, this means that the choice between NBI and high-definition WLI may be guided more by lesion characterization than by improved detection.

NBI has been previously shown to enhance the detection of gastric lesions, as highlighted by Dinis-Ribeiro et al. [4,29]. Yao et al. [30] also reported that magnifying NBI achieved exceptional diagnostic accuracy, with specificity reaching up to 99.4% for the detection of early gastric cancer within their study cohort.

Generally, the incidence of synchronous or metachronous multiple gastric cancers in patients with gastric neoplasms has been reported to range between 6.7% and 14.5% [31,32,33], highlighting the clinical importance of assessing detection rates in this population. Notably, our study is the first meta-analysis to include trials using 3G-NBI, which offers enhanced brightness and image clarity compared with earlier systems. This integration distinguishes our work from previous reviews limited to first- and second-generation NBI studies, thereby providing a more up-to-date evaluation of NBI’s diagnostic role. This modality can be combined with Texture and Color Enhancement Imaging (TXI), as demonstrated in the study by Kadota et al. [26], to enhance mucosal visualization. However, due to the limited availability of comparator arms across the remaining RCTs, a subgroup analysis incorporating these advanced imaging combinations could not be performed.

In our meta-analysis, all included RCTs were conducted in Eastern populations, which are relatively homogeneous and have a higher prevalence of gastric cancer [34,35]. This epidemiological context likely contributed to the higher proportion of true-positive results observed across the studies.

However, certain limitations should be acknowledged. The lack of data from Western populations limits the generalizability of our findings. Moreover, broader implementation of NBI in Western settings may be constrained by factors such as lower disease prevalence and higher associated costs [13]. Nevertheless, as emphasized in the MAPS III guidelines, VCE is now recommended for routine use in clinical practice, particularly for surveillance of patients at risk of gastric neoplasia [4]. Furthermore, in cases of EGC with a lesion diameter less than 10 mm, accurate biopsy sampling is critical. The potential for false-negative histological results in such small lesions may introduce bias and contribute to diagnostic uncertainty [36,37]. Another important limitation is the inability of NBI to reliably assess the depth of tumor invasion. Consequently, some patients initially managed with endoscopic mucosal resection (EMR) or endoscopic submucosal dissection (ESD) may ultimately require surgical intervention based on post-resection histological findings. It is also important to consider that all procedures in the included studies were performed by experienced endoscopists specifically trained in NBI. According to the ESGE guidelines [4], such targeted training should be made available to all endoscopists involved in gastric cancer surveillance to ensure consistency and diagnostic accuracy. Beyond operator expertise, variability in pre-procedural preparation protocols may also impact diagnostic outcomes. Even within the same country, practices can differ—for instance, the administration of mucolytic agents such as simethicone prior to gastroscopy is employed in some settings to improve mucosal visibility and facilitate lesion detection [38,39].

In the existing literature, the reported sensitivity of NBI combined with ME ranges from 0.60 to 0.97 [30,40], reflecting variability across study designs. A network meta-analysis [41] further explored the diagnostic accuracy of various VCE modalities in detecting EGC, specifically evaluating the performance of ME when paired with different imaging techniques. In a pooled cohort of 5948 patients, ME was used in conjunction with NBI, BLI, and WLI; however, LCI was not included as a comparator. The analysis demonstrated that both ME-NBI and ME-BLI significantly outperformed ME-WLI in diagnostic accuracy, while there was no significant difference in performance between ME-NBI and ME-BLI. Nonetheless, the study had notable heterogeneity, particularly due to inconsistent application of optical magnification (ME) versus digital magnification, as well as a lack of standardized population criteria across the included studies.

Regarding the remaining VCE modalities, two RCTs have compared LCI to WLI [42,43], and one RCT has assessed BLI [44] in a high-risk population. Ono et al. [42] conducted a multicenter crossover RCT involving 1502 patients with a history of gastrointestinal cancer across 16 Japanese hospitals. Participants underwent both LCI and WLI, in alternating sequences. The study demonstrated a significantly higher GCDR with LCI compared to WLI (8.0% vs. 4.8%, *p* = 0.011). However, it is noteworthy that this cohort included patients with prior malignancies unrelated to the stomach or esophagus (for instance colorectal or pharyngeal cancer), which differs from the target population of our study. In another RCT conducted in China by Gao et al. [43], 2383 high-risk patients were randomized to undergo either LCI or WLI. The findings similarly showed a significantly higher GCDR in the LCI group compared to WLI (8.01% vs. 4.31%, *p* < 0.001). An additional RCT [45] evaluated LCI versus WLI in a general screening population undergoing upper gastrointestinal endoscopy, which does not align with the high-risk focus of our study. Nonetheless, within this screening cohort, the LCI group also demonstrated a significantly higher GCDR (*p* < 0.001). Dohi et al. [44] investigated BLI in a high-risk population for early gastric cancer, enrolling 629 patients from 2 Japanese hospitals. In this crossover RCT, patients underwent both BLI and WLI. The results indicated that BLI yielded a significantly higher GCDR compared to WLI (93.1% vs. 50.0%, *p* = 0.001).

A meta-analysis conducted by Zhang et al. [46] compared the diagnostic performance of ME-NBI to WLI in the detection of EGC. The analysis included 10 non-RCTs, of which 6 were prospective [7,30,40,47,48,49], encompassing a total of 1724 patients and 2153 lesions. A high degree of heterogeneity was noted among the studies, with six of the ten including lesions smaller than 10 mm—a known diagnostic challenge. The pooled results indicated that ME-NBI significantly improved the detection of EGC, yielding a sensitivity of 0.83 (95% CI: 0.79–0.87; I^2^ = 79.8%) and a specificity of 0.96 (95% CI: 0.95–0.97; I^2^ = 89.3%). Despite these encouraging findings, the authors acknowledged limitations due to the non-randomized nature of the included studies and the fact that only six were prospective in design, which may have introduced bias and affected the overall robustness of the conclusions. A more recent meta-analysis by Le et al. [41] included 8 prospective studies involving a total of 5948 patients and compared the diagnostic performance of ME combined with WLI, NBI, and BLI for the detection of EGC. The findings demonstrated that both ME-NBI and ME-BLI were significantly more accurate than magnifying WLI (ME-WLI), with odds ratios of 2.56 (95% CI: 2.13–3.13) and 3.13 (95% CI: 1.85–5.71), respectively. However, no statistically significant difference was found between ME-NBI and ME-BLI, suggesting comparable diagnostic performance between these advanced imaging modalities.

Rodríguez-Carrasco et al. [50] investigated the role of image-enhanced endoscopy (IEE), including NBI, in the detection of gastric pre-neoplastic and neoplastic lesions. For the neoplastic component, their analysis incorporated mostly prospective studies [30,47,48] rather than RCTs—only 1 [27]—focusing on dysplasia and EGC. While these studies contributed valuable insights, the inclusion of dysplasia alongside EGC introduced some heterogeneity in the study populations. Moreover, the study by Yamada et al. [51] specifically focused on the endoscopic features distinguishing depressed-type gastric cancer, rather than offering a direct comparative evaluation of imaging modalities. As a result, the analysis by Rodríguez-Carrasco et al. provided only indirect comparisons between NBI and WLI in the context of EGC detection.

Future research should prioritize large, multicenter RCTs in Western populations, where gastric cancer prevalence is lower and diagnostic challenges differ. Beyond prevalence, variability in training, endoscopy quality standards, and resource availability may affect the relative benefit of NBI. Another promising direction is the integration of NBI with artificial intelligence systems, which could help compensate for operator dependency and further reduce miss rates. Additionally, cost-effectiveness analyses are needed to assess whether adopting NBI as a routine modality is justified in different healthcare systems. Finally, international collaborations harmonizing endoscopy protocols, training curricula, and reporting standards would enhance the generalizability and implementation of findings across diverse settings.

## 5. Conclusions

This meta-analysis demonstrates that NBI offers a non-significant advantage over WLI in the detection of EGC among high-risk patients. Although the improvement in GNDR did not reach statistical significance, NBI remains a valuable tool in enhancing the visualization of mucosal and vascular patterns, particularly when combined with magnifying or NF technologies. Importantly, FGLDR and PPV were comparable between NBI and WLI, suggesting that while NBI may improve diagnostic confidence, its incremental benefit is context-dependent.

Given the growing incidence of EGC and its markedly higher survival rate when detected early, optimized endoscopic surveillance strategies are critical. The findings support the incorporation of high-definition endoscopy and VCE, including NBI, into routine clinical practice, especially in high-risk populations. Further large-scale RCTs with standardized protocols across diverse populations are required to validate these results and refine guidelines for surveillance and diagnosis of EGC. In particular, validation in Western populations—where gastric cancer prevalence is lower and endoscopic expertise may vary—will be essential before these findings can be broadly translated into clinical practice.

## Figures and Tables

**Table 1:** Study characteristics.

Author, Year	Recruitment Period	Site	Patients			Mean Age (Years)		Female (N)	
			Total	NBI	WLI	NBI	WLI	NBI	WLI
Ang et al., 2015 [27]	2012–2013	Asia-Pacific region	579	293	286	62.6	62.3	165	178
Yoshida et al., 2021 [28]	2014–2017	Japan	4523	2265	2258	70.6	70.6	491	505
Kadota et al., 2024 [26]	2021–2022	Japan	901	300	301	73	73	72	73

## Data Availability

All data extracted from included studies and the analytic code used for the meta-analysis are available from the corresponding author upon reasonable request.

## Abbreviations

The following abbreviations are used in this manuscript:

BLI	Blue Laser Imaging
ECG	Early Gastric Cancer
EMR	Endoscopic Mucosal Resection
ESD	Endoscopic Submucosal Dissection
FGLDR	Focal Gastric Lesion Detection Rate
GA	Gastric Atrophy
GNDR	Gastric Neoplasm Detection Rate
IM	Intestinal Metaplasia
LCI	Linked Color Imaging
ME	Magnifying Endoscopy
NBI	Narrow-Band Imaging
NF	Near Focus Magnification
PPV	Positive Predictive Value
RCT	Randomized Control Trial
VCE	Virtual Chromoendoscopy
WLI	Wight Light Imaging

## Appendix A

**Table A1:** Quality assessment—Risk of bias [26].

Bias	Authors’ Judgement	Support for Judgement
Random sequence generation (selection bias)	Low risk	“Patients were randomly assigned in a 1:1:1 ratio to 3G-NBI (primary 3G-NBI and secondary WLI), TXI (primary TXI and secondaryWLI), and WLI (primary and secondaryWLI) groups.”
Allocation concealment (selection bias)	Low risk	“The WLI group was set as the calibration arm by the minimization method with a random component balancing the groups regarding institution, age (<70 and >70 years), indication for endoscopy (surveillance and preoperative), and organ of previously treated or known lesion (stomach and oesophagus).”
Blinding of participants and personnel (performance bias)	High risk	“The endoscopists did not attempt to mask allocation to the study group.”
Blinding of outcome assessment (detection bias)	High risk	Blinding of investigators to the intervention method was not implemented, which may have introduced bias in outcome assessment due to the potential influence of unblinded observation.
Incomplete outcome data (attrition bias)	Low risk	The per-protocol analysis was performed following the exclusion of comparable numbers of patients from each of the three allocation arms, based on consistent exclusion criteria. “After randomization, 5 patients were excluded: 2 were ineligible, one violated the study protocol, and 2 had esophageal stenosis.”
Selective reporting (reporting bias)	Unclear risk	The protocol for the study was accessible, and the key pre-specified outcomes of interest to this review were reported in accordance with the original study plan.

**Table A2:** Quality assessment—Risk of bias [27].

Bias	Authors’ Judgement	Support for Judgement
Random sequence generation (selection bias)	Low risk	“Patients were randomized in a 1: 1 ratio in blocks of 20 to undergo either NBI or HD-WLE for UGI endoscopy.”
Allocation concealment (selection bias)	Low risk	“Individual random sequence was placed in an opaque envelope and kept by an independent research assistant who was not involved in this study.”
Blinding of participants and personnel (performance bias)	High risk	The authors did not specify whether participants were informed about their allocation group. Regarding personnel blinding, the authors made the following statement: “Once informed consent was obtained, the research assistant would disclose the assigned imaging technique (NBI or HD-WLE) to the responsible endoscopist immediately before the procedure.”
Blinding of outcome assessment (detection bias)	High risk	The investigators were not blinded to the intervention method, which may have introduced bias in outcome assessment due to the lack of blinding.
Incomplete outcome data (attrition bias)	Low risk	Per protocol analysis performed after exclusion of balanced proportion of patients with similar reasons between the two allocation groups. “A total of 600 patients were initially enrolled, but as 21 patients were actually younger than 50 years of age and thus outside recruitment criteria, they were excluded, leaving 579 patients for analysis.”
Selective reporting (reporting bias)	Unclear risk	The study protocol is available, and the primary pre-specified outcomes relevant to this review—with the exception of the positive predictive value—were reported as originally planned.

**Table A3:** Quality assessment—Risk of bias [28].

Bias	Authors’ Judgement	Support for Judgement
Random sequence generation (selection bias)	Low risk	“Patients were randomly assigned in a 1:1 ratio to the WLI group (primary WLI followed by secondary 2G-NBI) or the 2G-NBI group (primary 2G-NBI followed by secondary WLI). A centralised randomisation process was conducted using a computerised minimisation procedure on the Medical Research Support Web site (Kyoto, Japan).”
Allocation concealment (selection bias)	High risk	Authors do not report the exact method of allocation concealment.
Blinding of participants and personnel (performance bias)	High risk	Blinding of study group assignments was not implemented for either the endoscopists or the patients.
Blinding of outcome assessment (detection bias)	High risk	Investigators were aware of the intervention used, which may have introduced bias in the assessment of outcomes due to the lack of blinding.
Incomplete outcome data (attrition bias)	Low risk	Per protocol analysis performed after exclusion of balanced proportion of patients with similar reasons between the two allocation groups. “After randomisation, 51 patients were excluded: 21 patients who had inadequate preparation; 15 patients who violated the study protocol; 4 patients who had intragastric haemorrhage; 4 patients who had oesophageal stenosis; and 7 patients who had other reasons.”
Selective reporting (reporting bias)	Unclear risk	A publicly accessible protocol was provided, and the main outcomes of interest to this review were reported as originally specified.

**Table A4:** Quality of body evidence—Summary of Findings Table (GRADE).

Participants (Studies)	Risk of Bias	Inconsistency	Indirectness	Imprecision	Publication Bias	Other Considerations	Overall Certainty of Evidence	Study Event Rates (%)		Relative Effect (95% CI)	Anticipated Absolute Effects	
								Risk with WLI	Risk with NBI		Risk with WLI	Risk Difference with NBI
^GNDR^ 5703 (3 RCTs)	serious	serious	not serious	not serious	undetected	none	⊕⊕⊝⊝ Low	441/2845 (15.5%)	421/2858 (14.7%)	RR 1.05 (0.72 to 1.52)	155 per 1000	8 more per 1000 (from 43 fewer to 81 more)
^FGLDR^ 5703 (3 RCTs)	serious	serious	not serious	not serious	undetected	none	⊕⊕⊝⊝ Low	78/2845 (2.7%)	80/2858 (2.8%)	RR 0.98 (0.66 to 1.45)	27 per 1000	1 fewer per 1000 (from 9 fewer to 12 more)
^PPV^ 773 (2 RCTs)	serious	serious	not serious	not serious	undetected	none	⊕⊕⊝⊝ Low	88/428 (20.6%)	102/345 (29.6%)	RR 1.29 (0.84 to 1.99)	206 per 1000	60 more per 1000 (from 33 fewer to 204 more)
